# Multiple introductions and population structure during the rapid expansion of the invasive Sahara mustard (*Brassica tournefortii*)

**DOI:** 10.1002/ece3.5239

**Published:** 2019-06-28

**Authors:** Daniel E. Winkler, Kenneth J. Chapin, Olivier François, J. David Garmon, Brandon S. Gaut, Travis E. Huxman

**Affiliations:** ^1^ Department of Ecology and Evolutionary Biology University of California, Irvine Irvine California; ^2^ U.S. Geological Survey Southwest Biological Science Center Moab Utah; ^3^ Department of Ecology and Evolutionary Biology University of California, Los Angeles Los Angeles California; ^4^ Department of Ecology and Evolutionary Biology University of Arizona Tucson Arizona; ^5^ Université Grenoble‐Alpes Grenoble France; ^6^ Tubb Canyon Desert Conservancy Borrego Springs California

**Keywords:** desert, genetic diversity, genotyping by sequencing, nextRAD, SNP, southwest

## Abstract

The specific mechanisms that result in the success of any species invasion case are difficult to document. Reproductive strategies are often cited as a primary driver of invasive success, with human activities further facilitating invasions by, for example, acting as seed vectors for dispersal via road, train, air, and marine traffic, and by producing efficient corridors for movement including canals, drainages, and roadways. Sahara mustard (*Brassica tournefortii*) is a facultative autogamous annual native to Eurasia that has rapidly invaded the southwestern United States within the past century, displacing natives, and altering water‐limited landscapes in the southwest. We used a genotyping‐by‐sequencing approach to study the population structure and spatial geography of Sahara mustard from 744 individuals from 52 sites across the range of the species’ invasion. We also used herbaria records to model range expansion since its initial introduction in the 1920s. We found that Sahara mustard occurs as three populations in the United States unstructured by geography, identified three introduction sites, and combined herbaria records with genomic analyses to map the spread of the species. Low genetic diversity and linkage disequilibrium are consistent with self‐fertilization, which likely promoted rapid invasive spread. Overall, we found that Sahara mustard experienced atypical expansion patterns, with a relatively constant rate of expansion and without the lag phase that is typical of many invasive species.

## 1 INTRODUCTION

Successful invasions often occur when the dispersal barriers that prevent species movement break down (van Kleunen, Dawson, & Maurel, 2015; Mooney & Cleland, 2001; Wilson, Dormontt, Prentis, Lowe, & Richardson, 2009). Following the loss of dispersal barriers, invasives spread and often establish separate geographic populations. By elucidating spatial genetic patterns, one can gain insights into the ecological and evolutionary mechanisms that have enabled the success of invasives (Colautti & Lau, 2015; Cristescu, 2015; Lee, 2002). In particular, understanding the population structure of invasive species can provide insight into the history of dispersal during the colonization and expansion process (Barker, Andonian, Swope, Luster, & Dlugosch, 2017; Durka, Bossdorf, Prati, & Auge, 2005; Eriksen et al., 2014; Peccoud et al., 2008). For example, multiple introductions of the invasive yellow starthistle (*Centaurea solstitialis*) in North America were identified by examining the species’ population genetics across its invaded range (Barker et al., 2017; Dlugosch, Lai, Bonin, Hierro, & Rieseberg, 2013; Sun, 1997). Similarly, population structure analyses revealed that multiple introductions of the invasive pea aphid (*Acyrthosiphon pisum*) allowed it to colonize separate host plant species across South America (Peccoud et al., 2008). Population genetic studies of invasive species have often revealed that a mixed set of processes dictate population structure, making it difficult to find a general suite of successful invasive characteristics (Sakai et al., 2001; Simberloff et al., 2013).

Although a variety of characteristics have been used to explain successful invasive species establishment and spread, reproductive strategies are often cited as a primary driver among plants (Burrell et al., 2015; van Kleunen et al., 2015; Richards, Bossdorf, Muth, Gurevitch, & Pigliucci, 2006; Sakai et al., 2001). Indeed, self‐compatibility and other flexible reproductive strategies (e.g., vegetative propagation, apomixis) are common in invasive plant species (Baker, 1955, 1967; Colautti et al., 2005; Dlugosch & Parker, 2008; Pannell et al., 2015; Pappert, Hamrick, & Donovan, 2000). These reproductive systems enable populations to persist and spread from only one or a few individuals (Blackburn, Lockwood, & Cassey, 2015; Cheptou, 2004; Dornier, Munoz, & Cheptou, 2008; Schoen, Morgan, & Bataillon, 1996). Nonetheless, invading species typically undergo an initial lag phase where populations remain small before a relatively sudden range expansion (Bock et al., 2015; Pannell, 2015); the species’ breeding system may determine the length of the lag or ameliorate it altogether (Crooks, 2005; Crooks & Soulé, 1999; Parker, 2004).

Invasions often arise from multiple introduction events. Subsequent admixture can increase invasion success (Dlugosch & Parker, 2008; Durka et al., 2005; Hahn & Rieseberg, 2017; Lombaert et al., 2010) by increasing genetic diversity, thereby decreasing inbreeding depression and potentially enabling adaptation (Barker et al., 2017; Dlugosch, Anderson, Braasch, Cang, & Gillette, 2015; Lavergne & Molofsky, 2007; Lawson Handley et al., 2011; Parker, Rodriguez, & Loik, 2003; Peischl & Excoffier, 2015; Prentis, Wilson, Dormontt, Richardson, & Lowe, 2008; Rius & Darling, 2014). However, there is evidence that not all invasive species experience the negative effects of reduced genetic diversity when initial founder populations are large (Holle & Simberloff, 2005; Roman & Darling, 2007) or when reproductive assurance is provided by self‐fertility (Daehler, 1998; Schoen et al., 1996). Varied scenarios like these may explain why lag phases range from nearly no delay to over 300 years (Crooks & Soulé, 1999). For example, 197 of 257 datasets on invasive species in the Midwest region of the United States exhibited clear lags that ranged from 3 to 140 years while the remaining species showed no sign of a lag phase during the invasion process (Larkin, 2012). These scenarios also highlight that invasion success is in part determined by the standing genetic variation of one or multiple introductions (Estoup & Guillemaud, 2010; Kolbe et al., 2004).

Sahara mustard (*Brassica tournefortii*; Family: Brassicaceae) is a facultative autogamous (i.e., primarily self‐fertilizes but outcrossing is possible) diploid annual that is native to the Mediterranean basin and much of the Middle East into western India (Aldhebiani & Howladar, 2013; Prain, 1898; Thanos, Georghiou, Douma, & Marangaki, 1991). It is a pest species in agriculture fields in parts of its native range and Australia (Ahmed, Fawzy, Saeed, & Awad, 2015; El‐Saied, El‐Ghamry, Khafagi, Powell, & Bedair, 2015; Salisbury, Potter, Gurung, Mailer, & Williams, 2018), but it also has traditional dietary uses and economic value in regions where it is cultivated (Guarrera & Savo, 2016; Singh, Semwal, & Bhatt, 2015). Sahara mustard is an invasive throughout much of Australia (Chauhan, Gill, & Preston, 2006), South Africa (McGeoch, Kalwij, & Rhodes, 2009), Chile (Teillier, Prina, & Lund, 2014), and more recently, western North America (Li, Dlugosch, & Enquist, 2015). It germinates under a wide range of temperatures, light, soil conditions, and depths (Bangle, Walker, & Powell, 2008; Chauhan et al., 2006; Jurado & Westoby, 1992; Thanos et al., 1991), and it produces seeds rapidly (ca. 50 days from germination; Marushia, Brooks, & Holt, 2012) and in high quantities (Trader, Brooks, & Draper, 2006). These seeds can remain viable at least 1 year after production (Chauhan et al., 2006) and can likely undergo some level of dormancy, similar to desert annuals with which it co‐occurs (Adondakis & Venable, 2004). The seeds contain a mucilaginous film that protect seeds from desiccation and is thought to allow for increased dispersal via roadways, animals, and water (Bangle et al., 2008). Its genome is approximately 791 Mbp (Arumuganathan & Earle, 1991) and is substantially divergent from even its most closely related relatives in the *Brassica* genus (Sánchez‐Yélamo, Ortiz, & Gogorcena, 1992).

The first documented occurrence of Sahara mustard in the United States comes from an herbarium sample collected near Palm Springs in the Coachella Valley, California in 1927. It may have been introduced as a contaminant of cultivated date palm (Sanders & Minnich, 2000) and remained confined to the Coachella and Imperial Valleys of the Sonoran and Mojave Deserts where it established locally (Musil, 1948, 1950; Robbins, Bellue, & Ball, 1951). Some authors hypothesized a population boom beginning in the 1980s when it spread rapidly throughout the southwest (Sanders & Minnich, 2000), potentially suggesting a lag phase had previously occurred. To date, Sahara mustard's introduction point(s) in the United States remains unknown. Still, Sahara mustard is having ever‐greater impacts on natural ecosystems across the southwestern United States (Barrows, Allen, Brooks, & Allen, 2009; VanTassel et al., 2014). Since its presumed introduction in the 1920s, this invader has become increasingly common in semi‐arid regions, including all counties in Southern California (Sanders & Minnich, 2000) and throughout 500,000 km^2^ ha in Southwest United States and Northwest Mexico. Although a few ecological studies have examined the species’ performance and impacts in a few invaded areas (Barrows et al., 2009; Li et al., 2015; Marushia et al., 2012; Marushia, Cadotte, & Holt, 2010; VanTassel et al., 2014; Winkler, Gremer, Chapin, Kao, & Huxman, 2018), no research has been conducted to examine the genetic structure of this invasive. Identifying introduction sites and understanding how the species has, and is, spreading via population genomics is a critical first step to elucidating the mechanisms by which species invasions can rapidly occur over large distances.

In this study, we use genotyping by sequencing to generate genome‐wide polymorphism data from across the invaded range of Sahara mustard in the western United States. We used these data along with historical distribution records to answer three questions: What is the current population structure of the species throughout its invaded range? What does population structure imply about the number of introductions and their locations? Has the geography and ecology of the western United States shaped the species distribution? Given anecdotal evidence and invasion studies of other species, we expected distribution records to reveal a lag phase as Sahara mustard established and spread in the United States. We also expected Sahara mustard to have low genetic diversity given that the species can self‐fertilize. We posit both that multiple introductions are likely and that population structure has been shaped by ecosystems across the invaded range.

## 2 MATERIALS AND METHODS

### 2.1 Historical range expansion

We utilized distribution and locality records from herbaria to examine the geographic spread of Sahara mustard in North America through time. We realize these data often provide an incomplete picture of a species range perhaps due to uneven collection efforts that do not accurately represent invasion patterns (Williamson, 2006). Nevertheless, distribution records can provide insights into invasion patterns and can be considered a conservative underestimate of range expansion (Crawford & Hoagland, 2009; Delisle, Lavoie, Jean, & Lachance, 2003). We obtained 2,834 records with collection dates and spatial data from three online databases: the Consortium of California Herbaria (http://ucjeps.berkeley.edu/consortium/), the Global Biodiversity Information Facility (http://www.gbif.org), and the Southwest Environmental Information Network (http//:swbiodiversity.org/seinet/index.php). We also included observation data from our field collections, bringing our total number of localities to 2,915.

We estimated the spread of Sahara mustard populations using distribution record locality data by counting the number of spatial units that the species occupied across time (1927–2016). Spatial units were delimited by rounding geographic degrees to the nearest hundredth. Thus, records were classed into ca. 1 km^2^ units across the invaded range. We created accumulation curves of the number of spatial units occupied by Sahara mustard per year. Ranges are expected to expand exponentially, but lag phases can alter this curve (Crawford & Hoagland, 2009; Crooks, 2005). As such, we tested for a lag phase by examining fit of a linear regression on log_10_ transformed cumulative range expansion, estimated by herbaria records, while acknowledging that these rates are likely conservative underestimates given the inherent biases often found in herbaria records (Delisle et al., 2003).

### 2.2 Sampling and genotyping

**Figure 1:**
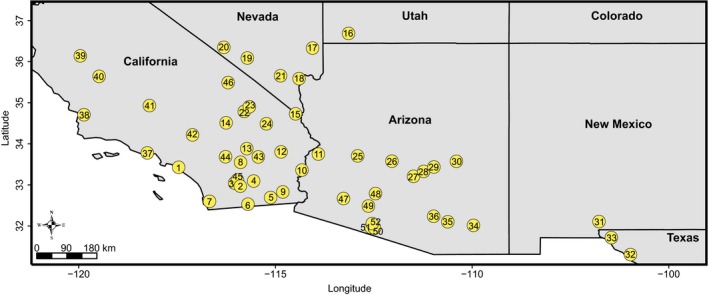
Map of sampling sites in California, Arizona, New Mexico, Texas, Nevada, and Utah. Numbers correspond to the following site names referenced in Table S1: (1) Irvine, (2) Anza1, (3) Anza2, (4) Salton City, (5) El Centro, (6) Ocotillo, (7) San Diego, (8) Coachella, (9) Glamis, (10) Blythe, (11) Parker, (12) JOTR1, (13) JOTR2, (14) MOJA1, (15) Ibis, (16) Leeds, (17) MormonPk, (18) LAKE1, (19) Amargosa, (20) Beatty, (21) Las Vegas, (22) MOJA2, (23) MOJA3, (24) MOJA4, (25) Aguila, (26) Phoenix1, (27) Phoenix2, (28) Tortilla Flat, (29) Roosevelt, (30) Fort Apache, (31) Las Cruces, (32) Fort Hancock, (33) El Paso, (34) Dragoon, (35) SAGU1, (36) SAGU2, (37) Malibu, (38) Nipomo, (39) Chaney Ranch, (40) Murray, (41) Bakersfield, (42) Victorville, (43) JOTR3, (44) Palm Springs, (45) Anza3, (46) DEVA, (47) Dateland, (48) Gila Bend, (49) Rocky Pt, (50) ORPI1, (51) ORPI2, (52) ORPI3

In Spring 2015, we sampled 7–20 (with an average of 14) individuals each from 52 locations (760 individuals total) spanning a ca. 10° latitudinal and ca. 15° longitudinal gradient across the species’ invaded U.S. range in Spring 2015 (Figure 1; Table S1). Sites ranged from coastal Mediterranean to hot desert ecosystems with elevation ranging 0–1500 m asl (Table S1). Tissue for genetic analyses was desiccated with silica gel for preservation.

We extracted DNA from 760 individuals from 52 populations using QIAGEN DNeasy Plant Mini Kits (Qiagen). We estimated DNA concentrations via fluorometry (Qubit 2.0 Fluorometer, Invitrogen, Life Technologies) and tested DNA quality for a subset of samples via 1% agarose gel electrophoresis. Single nucleotide polymorphism (SNP) data were generated via nextRAD (Nextera‐tagmented, reductively amplified DNA) sequencing (Russello, Waterhouse, Etter, & Johnson, 2015; libraries were prepared and sequenced by SNPsaurus, LLC). NextRAD uses short oligonucleotide primers to amplify arbitrary loci across genomic samples. Primers were integrated into the Nextera library preparation protocol (Illumina, Inc), which also ligates short adapter sequences to the ends of the DNA fragments. DNA fragments with one of the primers matching the adapter sequence were then amplified, and pooled samples were barcoded before purification and size selected from 350 to 500 bp. Multiplexed segments were sequenced on an Illumina HiSeq2000 platform (Genomics Core Facility, University of Oregon) producing 100 bp single read lengths.

Raw sequence data were processed using Trimmomatic software (Bolger, Lohse, & Usadel, 2014) to remove adapter sequences and filter sequences less than 50 bp. Sequences were quality‐filtered using the program process_radtags in STACKS (Catchen, Amores, Hohenlohe, Cresko, & Postlethwait, 2011; Catchen, Hohenlohe, Bassham, Amores, & Cresko, 2013). Sequences with at least 15–2,500× coverage and those that were present in at least 10% of samples were retained. To exclude paralogs, loci were removed if more than two alleles were found in a sample in more than 5% of a sampling locality (Hare, 2001; Russello et al., 2015). The remaining sequences were then mapped to a reference created using abundant reads across the combined set of samples using the program BBmap v.35.40 (http://sourceforge.net/projects/bbmap; sensu Russello et al., 2015). A total of 16 individuals were removed from the final dataset due to >75% missing data. In total, 1,525 SNPs were identified across the 744 sequenced individuals. We took 1,000 reads randomly from each sample for comparison to known sequences in the NCBI database via BLAST (https://blast.ncbi.nlm.nih.gov/Blast.cgi) to test for contamination from species other than Sahara mustard. No plausible contamination was detected in the tested reads.

### 2.3 Genomic analyses

We first estimated the number and location of genetic clusters using the spatial Bayesian clustering algorithm implemented in TESS 2.3.1 (Chen, Durand, Forbes, & François, 2007; Durand, Jay, Gaggiotti, & François, 2009). TESS uses spatial locations of samples to construct a neighborhood network of individuals to measure spatial patterns of genetic relatedness; a suitable method for selfing species compared to other population assignment programs (Fogelqvist, Niittyvuopio, Ågren, Savolainen, & Lascoux, 2010). We used the admixture model (CAR) set at the default spatial interaction parameter ψ = 0.6 with a burn‐in length of 10,000, a run length of 50,000, and performed 10 iterations of *k* = 2–10. Deviance information criterion was averaged and plotted for each *k* to select the optimum number of clusters (sensu Chen et al., 2007). We plotted mean membership scores per sampling site as admixture proportions following François (2016). We visualized mean membership of sample sites using the LEA R package (Frichot & François, 2015). We used the mean membership values from the ten TESS runs of the optimal *k* value to visualize admixture at each site by overlaying results onto a map of the sampling range.

As a compliment to TESS analyses, we visualized the population structure of sequenced individuals using a principal component analysis (PCA) that constructs population differentiation relationships (François et al., 2010; Gross, Hosoya, & Queloz, 2014; Ma & Amos, 2012). We then calculated the number of overall and per‐cluster rare variants (minor allele frequency <10%) to identify the most likely origins of clusters identified by TESS (Cubry, Vigouroux, & François, 2017). To accomplish this, we interpolated the density of rare variants on a map of the invaded area using a kriging approach without trend surface in the fields R package (Nychka, Furrer, & Sain, 2017) in order to estimate likely ancestral regions of Sahara mustard in the United States (Alvarado‐Serrano & Hickerson, 2018; Cubry et al., 2017). We also calculated the density of rare variants by site distance from the putative introduction sites using local regression in R.

We also grouped localities by ecoregions (Table S1) and used a hierarchical analysis of molecular variance (AMOVA) to estimate the variance within and between localities and ecoregions (Excoffier, Smouse, & Quattro, 1992). We estimated overall linkage disequilibrium with ${\bar{r}}_{d}$ (a measure of the index of association that accounts for sample size; Agapow & Burt, 2001) as an indicator of selfing (Ingvarsson, 2002; Nordborg, 2000). We calculated inbreeding coefficients (*F*_is_) for each site and to calculate population‐level selfing rates (*S* = 2*F*_is_/(1 + *F*_is_)) and levels of outcrossing (*T* = 1 − *S*; Hedrick, 2011; Wright, 1921). We calculated the number of private alleles in each population to examine levels of isolation between groups. Analyses were executed in R 3.3.2 with the adegenet, pegas, and poppr packages (Jombart & Ahmed, 2011; Kamvar, Tabima, & Grünwald, 2014; Paradis, 2010; R Core Team, 2014).

## 3 RESULTS

### 3.1 Historical range expansion

**Figure 2:**
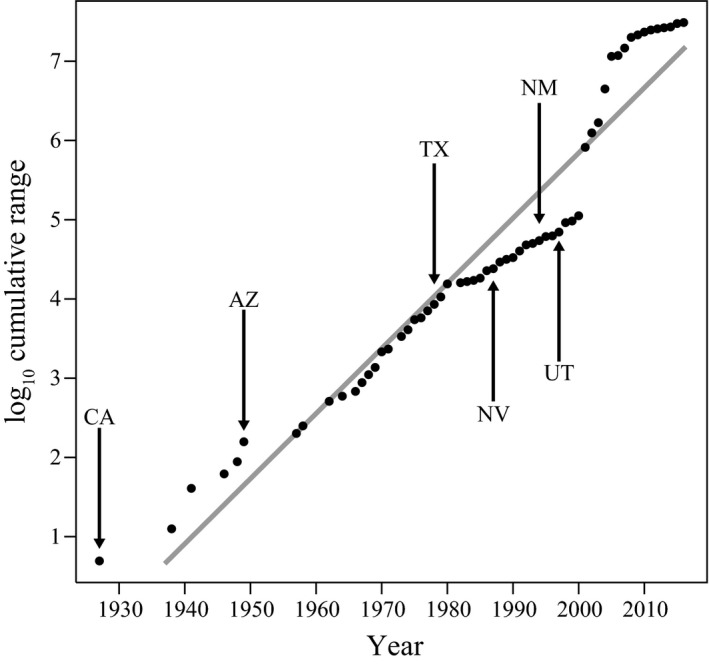
Estimated range expansion of Sahara mustard (*Brassica tournefortii*) using herbaria records with a linear regression of years (1927–2016) predicting the log_10_ cumulative observations of the species’ range in unique ca. 1 km^2^ areas (*b* = 0.0823, *R* ^2^ = 0.93, *F*_1,57_ = 808.1, *p* < 0.001). Arrows indicate the first occurrence of Sahara mustard in each state: California (CA), Arizona (AZ), Texas (TX), Nevada (NV), New Mexico (NM), and Utah (UT)

We used 2,915 historic and contemporary locality records to study the history of Sahara mustard's range expansion. By interpolating the year of sampling with its geographic range, we infer that Sahara mustard underwent an atypical invasion with no detectable lag phase and a relatively constant postintroduction expansion pattern (Figure 2). A linear regression of year predicting log_10_ cumulative range expansion showed excellent fit (*b* = 0.0823, *R* ^2^ = 0.93, *F*_1,57_ = 808.1, *p* < 0.001), consistent with the lack of a lag phase in the history of the invaded range. Herbaria records tracked the spread of Sahara mustard to Tucson, Arizona in the late 1940s, coastal California in the late 1950s, and more recently to Texas, Nevada, New Mexico, and Utah. Overall, Sahara mustard's range based on sampling sites currently stretches approximately 500,000 km^2^ based on herbaria records and our field sampling (Figure 1). This is an underestimate of the species range given that sampling was carried out in 2015 for this current analysis, and expansion has likely occurred since then. The most dramatic change occurred as Sahara mustard was identified as a management concern—especially after 2000. Overall observed expansion patterns have slowed since 2010 and might be reaching a stable distribution, perhaps due to environmental constraints. However, the apparent slowing of expansion could be caused by collection effort biases (Figure 2; Video S1; Cousens & Mortimer, 1995), due to rapid roadway sampling that occurred in 2004–2005 (https://www.cal-ipc.org/solutions/research/saharan/).

### 3.2 Population structure

**Figure 3:**
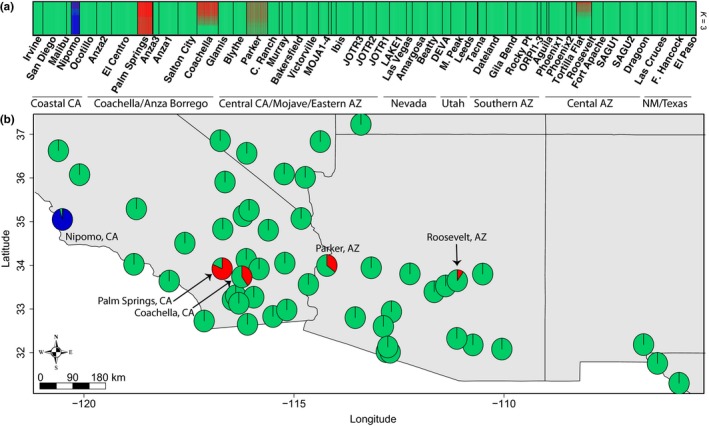
(a) Individual assignments from TESS analyses based on 1,525 SNP loci of 744 individuals from across the invaded range of Sahara mustard (*Brassica tournefortii*). Barplots are averaged across ten runs of the highest likely number of clustered predicted to be *K* = 3. (b) Map illustrating average assignment probabilities to each cluster from TESS analyses (pie chart colors: Cluster 1 = green, Cluster 2 = red, and Cluster 3 = blue). Clusters 2 and 3 are named by sampling site. Site names correspond to those referenced in Table S1

We gathered 744 plants from 52 locations and identified 1,525 SNPs that we analyzed with the program TESS, in order to identify potential population structure. TESS analyses revealed population structure across the invaded range of Sahara mustard, with three genotypic clusters (Figures 3a and S1). Cluster 1 included plants from across most of the species invaded range; Cluster 2 was focused on Palm Springs in the Coachella Valley but also included isolates from Coachella, CA, Parker, AZ, and Roosevelt, AZ; Cluster 3 was limited to Nipomo, CA. TESS also identified apparent admixture occurring between Clusters 1 and 2. Individuals from Palm Springs, CA had the highest probability of assignment to Cluster 2, but the Coachella, CA, Parker, AZ, and Roosevelt, AZ sites also had some significant probability of assignment to this cluster (Figure 3a). Overall, TESS analyses revealed that three genetically distinct populations exist in the United States based on the sites we sampled but that some individuals exhibited multiple assignment to Clusters 1 and 2. This was evidenced by replicates of each *K* value separating Clusters 2 and 3 sites from Cluster 1 across all runs of *K* (Figure S1). The mean log probability of the data increased with the successive addition of clusters to *K* = 3, after which it plateaued. Cluster 3 contained 303 private alleles that were found nowhere else in the invaded range while Cluster 1 had only 40 private alleles and Cluster 2 had 1 (Table 1), suggesting levels of isolation between populations vary.

**Table 1:** Summary statistics for each cluster as defined by TESS results

Population	*n*	*H*_o_ (±*SE*)	*H*_e _(±*SE*)	*F*_is_	*S*	*T*	Private
Cluster 1	668	0.0577 (±0.0065)	0.0697 (±0.0041)	0.8425	0.9145	0.0855	40
Cluster 2	68	0.0565 (±0.0066)	0.0562 (±0.0039)	0.7827	0.8778	0.1222	1
Cluster 3	12	0.0551 (±0.0066)	0.0356 (±0.0038)	0.7865	0.8805	0.1195	303

Note

*n* = number of individuals analyzed, *H*_o_ (±*SE*) = the observed heterozygosity for SNPs, *H*_e_ (±*SE*) = the expected heterozygosity for SNPs, *F*_is_ = index of fixation, *S* = selfing rate, *T* = outcrossing rate, and private = the number of private alleles. Standard errors are reported parenthetically.

Mapping the mean TESS assignment probabilities (= admixture coefficients) revealed no clear spatial patterns across the invaded range (Figure 3b). Cluster 1 was dominant throughout the entire invaded range and also occurred within sampling sites primarily assigned to the other clusters. The Nipomo, CA site was the only one with individuals assigned to Cluster 3. That being said, ca. 3% of individuals also exhibited multiple assignment with Cluster 1 (Figure 3b). This was a similar pattern for the Cluster 2 sampling sites but with varying degrees of assignment probabilities. The Palm Springs, CA site had the highest assignment probability to Cluster 2, followed by Coachella, CA, Parker, AZ, and Roosevelt, AZ exhibiting declines in Cluster 2 assignments as geographic distance increased from Palm Springs. Given this, the presumed Coachella Valley introduction site (Sanders & Minnich, 2000) is likely identified as Cluster 2, with a shift in genetic identity toward the more widespread genotype seen in Cluster 1.

**Figure 4:**
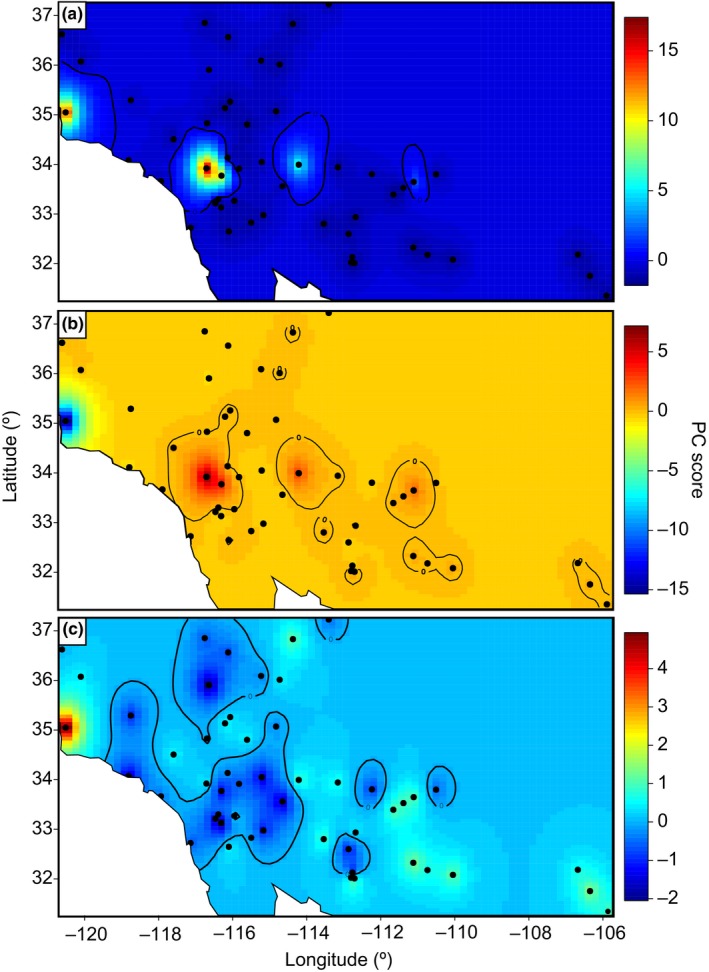
Principal component analysis (PCA) maps of Sahara mustard (*Brassica tournefortii*) in its invaded range. (a) PC1 highlights the largest population (= lowest, blue‐colored sites) and earliest introduction of Sahara mustard in the United States. (b) PC2 shows the grouping of the second introduction around Palm Springs in the Coachella Valley of California. (c) PC3 shows the most recent introduction restricted to Nipomo, CA

PCA revealed similar population structure across the invaded range of Sahara mustard but suggested more admixture than TESS (Figure 4). PC1 highlighted Cluster 1 (16.8% of variance; Figure 4a) and PC2 clearly identified Cluster 2 (7.4% of variance; Figure 4b). Lastly, PC3 identified the most divergent site: Nipomo, CA (Cluster 3; 5.3% of variance; Figure 4c). The AMOVA attributed most of the genetic variance to within‐locality variation, but variance between localities was also significant, indicating some population structure (Table 2). Despite this, practically no variance was explained by ecoregion (Table S1), consistent with our TESS results (Table 2).

**Table 2:** Hierarchical analysis of molecular variance (AMOVA) of *Brassica tournefortii* in the North American invaded range

	σ	% variance	ɸ	*p*
Within localities	59.68	74.88	0.05	<0.001
Between localities	16.12	20.23	0.21	<0.001
Between regions	3.90	4.89	0.05	0.094

Note

Most variance occurs within localities, while ecoregions explain almost no variance, indicating a lack of selection pressure across the invaded range.

**Figure 5:**
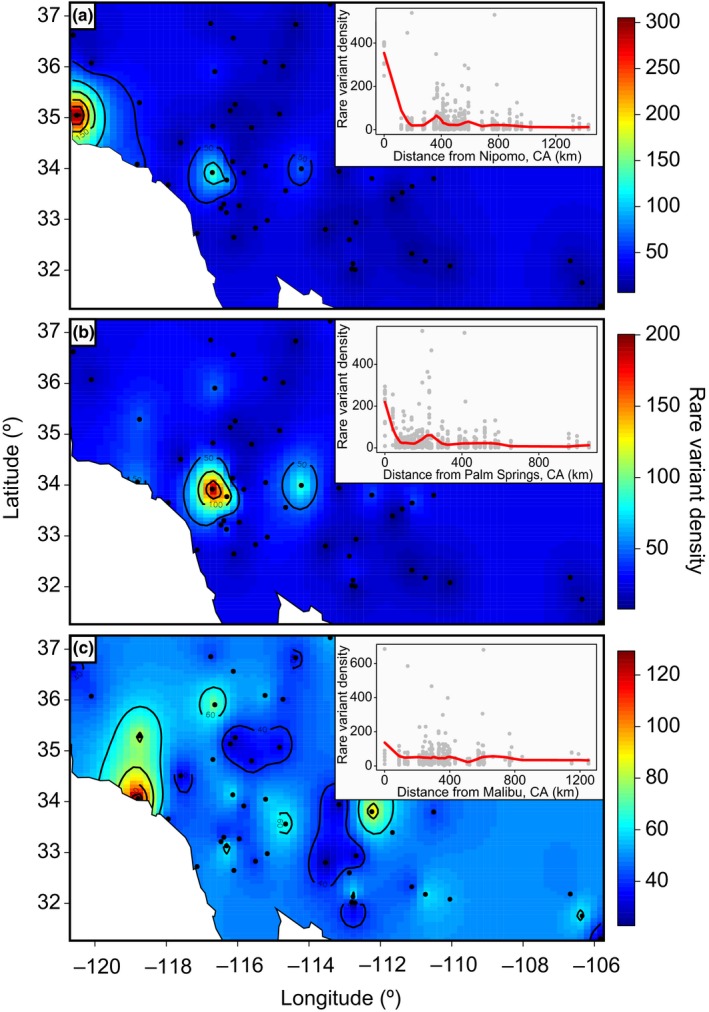
Density of rare variants indicating introduction sites in the invaded range of Sahara mustard (*Brassica tournefortii*). (a) Complete dataset highlighting Nipomo, CA as the most recent introduction site, with a decrease in rare variant density with distance from Nipomo (inset); (b) excluding the Nipomo, CA variants reveals Palm Springs in the Coachella Valley of California as the most likely origin for Cluster 2, with a decrease in rare variant density with distance from Palm Springs, CA (inset); (c) excluding all but the largest population (Cluster 1) highlights Malibu, CA as the most likely introduction point, with rare variant density generally decreasing with distance from Malibu (inset)

Nipomo, CA (Cluster 3) contained the highest number of rare variants which, combined with assignment probabilities from TESS, suggested that this is the most recent introduction of Sahara mustard and has yet to spread out of this area (Figure 5a). Removing Nipomo's rare variants from the PCA suggested a second introduction with a likely origin for Cluster 2 near Palm Springs, CA in the Coachella Valley (Figure 5b). Individuals from this site had a high number of rare variants but ca. 25% less than observed in Nipomo, CA (Figure 5b). Consistent with TESS results, the PCA showed admixture among Clusters 1 and 2. Further, genes from Cluster 2 propagated over relatively long distances into Arizona (Figure 5b). However, which sites contained admixed individuals differed slightly between TESS and the PCA but both analyses suggested sites in the Coachella Valley (Palm Springs and Coachella) share ancestry with Parker, AZ (Figures 3 and 5). Removing Cluster 2 rare variants revealed the most likely original introduction point for Cluster 1 was near Malibu, CA because the density of rare variants generally decreased with geographic distance from the Malibu site (Figure 5c). This is further supported by a lack of admixture in Cluster 1.

### 3.3 Genetic diversity and selfing

Linkage disequilibrium and overall genetic diversity were low across the invaded range of Sahara mustard (${\bar{r}}_{d}$ = 0.120, *p* = 0.009; Table S2). Further, nearly all sites had slightly lower levels of heterozygosity than expected but this was not the case at the population level (Table 1). Cluster 1 exhibited the highest levels of inbreeding (*F*_is_ = 0.8425) and selfing (*S* = 0.9145), and the lowest levels of outcrossing (*T* = 0.0855). Cluster 2 exhibited lower levels of inbreeding (*F*_is_ = 0.7827), selfing (*S* = 0.8778), and higher levels of outcrossing (*T* = 0.1222) than Cluster 1. Cluster 3 exhibited similar levels of fixation (*F*_is_ = 0.7865), selfing rates (*S* = 0.8805), and outcrossing rates (*T* = 0.1195) to Cluster 2; suggesting Clusters 2 and 3 are utilizing more of a mixed breeding system of self‐fertilization and outcrossing. That being said, all three populations appear to overwhelmingly self‐fertilized (Table 1).

## 4 DISCUSSION

Our study suggests Sahara mustard exists as three populations in the United States. The rapid spread and low genetic diversity of Sahara mustard are likely promoted by self‐fertilization, consistent with the observed expansion patterns and estimated selfing rates. The unusual spatial structure of non‐native Sahara mustard populations is most consistent with multiple introductions at Nipomo, Palm Springs, and Malibu, CA. Our work also suggests that subsequent range expansions have resulted in admixture among populations, which contributes to complex population structure. Despite these multiple inferred introductions, genetic diversity is generally low throughout the sampled locations and ecoregions. Low genetic diversity is likely the result of high self‐fertilization rates combined with founder effects, as suggested by other studies that have found self‐fertilization to be an important trait for colonization and rapid population expansion (Kalisz, Vogler, & Hanley, 2004; Levin, 2010; Lott, Volin, Pemberton, & Austin, 2003). We also showed that the invasion had no major lag phase, which surely accelerated its spread across the US Southwest (Crooks, 2005). Overall, we showed that Sahara mustard was most likely first introduced in the Malibu, CA area, with subsequent, independent introductions near Palm Spring, CA in the Coachella Valley and, most recently, near Nipomo, CA. Taken together, our population genomics analyses suggest that reproductive strategies and multiple introductions enabled Sahara mustard to colonize the diverse range of habitats despite the evolutionary roadblocks common to most invasions (Hargreaves & Eckert, 2014).

The reproductive strategies invasives employ are a primary driver of invasive success (Baker, 1955; Richards et al., 2006; Sakai et al., 2001). In this case, it is possible that mixed breeding systems within a species, in particular Sahara mustard's facultative autogamy, can speed the spread of invasive species by reducing inbreeding and other negative consequences that would normally affect obligate selfing species during colonization (Ansell, Grundmann, Russell, Schneider, & Vogel, 2008; Arnaud‐Haond et al., 2006; Daehler, 1998; Morgan, Wilson, & Knight, 2005; Saltonstall, 2003). Our results are consistent with the ability of a self‐fertilizing plant to rapidly expand its invaded range within decades. Self‐fertilization reduces the role of biotic interaction (i.e., does not require pollinators or sexual partners) and can promote establishment and spread (Baker, 1967; Barrett, Colautti, & Eckert, 2008; Pannell, 2015; Pannell et al., 2015). For example, *Spartina alterniflora* are cross‐pollination limited in the San Francisco Bay area of California and individuals that have high selfing rates also produce high viable seed sets compared to nonselfing individuals (Daehler, 1998). This is similar to patterns we observed in our current study and matches previous work showing Sahara mustard aligns its reproductive efforts based on ecological site factors (Winkler et al., 2018).

Invasive species traits and their relative importance oftentimes vary as a non‐native species goes through the phases of introduction, establishment, and spread (Bock et al., 2015; Bock, Kantar, Caseys, Matthey‐Doret, & Rieseberg, 2018; Hodgins, Bock, & Rieseberg, 2018; Pannell, 2015; Winkler et al., 2018) but reproductive strategies remain important throughout (Sakai et al., 2001). Self‐fertilization coupled with high propagule pressures can further reduce the potential negative impacts of inbreeding by increasing chances of establishment (Hargreaves & Eckert, 2014; Levin, 2010). A single Sahara mustard plant invests heavily in reproductive structures, can produce over 16,000 seeds, and disperses across relatively large distances via animals, wind, water, and roadways (Bangle et al., 2008; Berry, Gowan, Miller, & Brooks, 2014; Sánchez‐Flores, 2007; Trader et al., 2006; Winkler et al., 2018). It is likely that even if a small number of Sahara mustard were initially introduced into the United States, the species’ huge reproductive investments in offspring (Winkler et al., 2018) enable it to maintain adequate population sizes to overcome bottlenecks and establish itself at least locally (Lockwood, Cassey, & Blackburn, 2005). We observed 87%–91% selfing rates which are similar to results obtained in studies on other invasive plants (Kleunen, Fischer, & Johnson, 2007; Lott et al., 2003). Further, our analyses of herbaria records corroborate this by suggesting that a self‐compatible species is capable of avoiding the typical lag phase most invasives experience (Crooks, 2005; Crooks & Soulé, 1999; Parker, 2004). Expansion load, or the accumulation of deleterious mutations during range expansion, can prevent species from colonizing new environments if local adaptation has not occurred (Gilbert et al., 2017; Peischl & Excoffier, 2015). However, self‐fertilization can overcome these potentially expansion‐halting effects depending on the severity of the abiotic filters associated with establishment at a given site and inbreeding effects (Hamilton, Okada, Korves, & Schmitt, 2015; Hargreaves & Eckert, 2014; Pannell et al., 2015). Additionally, phenotypic plasticity may play a role in the species success across such broad environmental gradients (Richards et al., 2006), though no studies to date have identified plastic versus phenotypic variation. However, previous studies have revealed Sahara mustard occupies a wide breadth of environmental gradients and persists likely as a result of its ability to align various phenological, physiological, and morphological strategies across a broad range of environmental conditions (Winkler et al., 2018). A fruitful future direction should investigate whether self‐fertilization may have helped Sahara mustard overcome the severe environmental gradients it encounters across in the United States.

Attempts have been made to predict Sahara mustard's presence and abundance under future climate scenarios (Curtis & Bradley, 2015). Conservative models predict a considerable decline in suitable habitat, while less conservative models predict continued expansion (Curtis & Bradley, 2015). However, these models were limited by herbaria records and survey data that are biased by survey effort and timing and, in some cases, are not representative of the full extent of Sahara mustard distribution in the United States (Delisle et al., 2003; Williamson, 2006). Li et al. (2015) found that environmental variables of the native and invasive range of Sahara mustard are similar, suggesting that the species has not adapted to novel environments. However, these conclusions assume that the full suite of adapted genotypes from the native range was introduced in the invaded range. In fact, Sahara mustard natively occurs across a diverse geographic range that includes much of the Mediterranean basin and the Middle East into western India (Aldhebiani & Howladar, 2013; Prain, 1898; Thanos et al., 1991). Given that introduced species are often representative of a small regional population from the native range (Barker et al., 2017; Dlugosch & Parker, 2008; Lombaert et al., 2010), it is unlikely that the founding population would be perfectly adapted to the diversity of ecosystems in the invaded range. Our study reveals that Sahara mustard may have been introduced multiple times to California, which is particularly threatening to native systems, as admixture can produce novel genotypes, which might promote range expansion if it were to occur (Hahn & Rieseberg, 2017).

We generally detected low levels of genetic diversity across the invaded range of Sahara mustard; a pattern similar to other invasive species studied including those with mixed‐mating systems (i.e., Lott et al., 2003) and clonal species (Pappert et al., 2000; Sakai et al., 2001). This was consistent with our expectation, since facultatively self‐fertilizing species like Sahara mustard should experience reduced genetic diversity via reduced effective recombination and increased homozygosity (Charlesworth, 2003). Samples from the site at Nipomo, CA, however, showed considerable divergence from the rest of the range. This was likely caused by a recent introduction of Sahara mustard sometime after the second, Coachella Valley introduction (sensu Chen, Opp, Berlocher, & Roderick, 2006). If this is the case, the population in Nipomo, CA should receive high priority for eradication given that it is in initial invasion stages pre‐expansion. Further, the population has potential to hybridize with the other populations that, from experience with other invaders (e.g., Barker et al., 2017; Suarez & Tsutsui, 2008), could enable further range expansion. As is, the Nipomo population appears to be relatively isolated but should still be treated with concern given that humans are likely facilitating the species dispersal (Berry et al., 2014; Sánchez‐Flores, 2007; Trader et al., 2006). However, it is also possible that multiple genotypes were introduced in and around Nipomo, CA and only a subset was able to spread (Dlugosch & Parker, 2008; Lombaert et al., 2010). Further, we detected a decreasing number of rare variants with distance from the each of the presumed introductions in California. Rare variants have often been used to infer gene flow, migration, and connectivity of populations (Cubry et al., 2017; Genton, Shykoff, & Giraud, 2005; Pappert et al., 2000; Slatkin, 1985; Walker, Hulme, & Hoelzel, 2003); and our results suggest that a radiation away from Malibu and out of the Coachella Valley has occurred and also suggest isolation occurring at sites where the number of private alleles is high (Rollins, Woolnough, Wilton, Sinclair, & Sherwin, 2009; Verhoeven, Macel, Wolfe, & Biere, 2011). These sites should be targeted as high priority for land managers as they may enable localized control of these populations and could prevent future mixing with other populations (Rollins et al., 2009).

Sahara mustard has a similar invasion history in Australia where it was introduced in the early 1900s and is presumed to have dispersed via the transcontinental railroad (Kloot, 1987). The first record of Sahara mustard in the United States dates back to 1927 (Sanders & Minnich, 2000). Additional records of the species were relatively infrequent and concentrated to the deserts of southern California until around the 1970–1980s when it began appearing in neighboring states. Although the herbaria records we analyzed are intrinsically a subset of the actual occurrences of Sahara mustard, they suggest the species did not undergo a typical lag phase and, instead, was able to expand its range at a somewhat constant rate after its introduction, likely promoted by the species’ breeding system. That being said, lag phases are identified by slow range expansion early in the introduction; it could be that Sahara mustard is in the midst of a lag phase, in which case we expect rapid and wide expansion to occur in the western United States, given its prelag success. Since humans are facilitating the spread of Sahara mustard in the United States, it is unsurprising that the diversity has remained low across such a large range and that there are no clear genetic separations between populations. This low level of genetic diversity is the expected result of self‐fertilization coupled with human‐mediated dispersal. We expected population structure via vicariance or environmental variation but found that population structure seems to have been more affected by dispersal patterns. This includes human‐mediated dispersal, particularly roadways promoting long‐distance travel of seeds (Berry et al., 2014; Sánchez‐Flores, 2007; Trader et al., 2006). Our results are consistent with this dispersal mode, as evidenced by the species’ expansion from the Coachella Valley region of CA to sites as far away as Parker and Roosevelt, AZ (Figure 3).

In summary, our study is the first to document the genetic patterns of Sahara mustard's invasion in the United States and reveals the species exists as three populations with low levels of diversity—likely the result of self‐fertilization, combined with human‐mediated dispersal. The native range origins of these introductions remain unidentified. Future research is needed that will apply similar population genetic methods in the species’ native range to identify source populations and reconstruct the species’ invasion history globally. It will also be valuable to investigate the genetics of herbarium samples in the invaded ranges of the species to better understand invasion dynamics of this species. Successful management efforts will likely be achieved if human‐mediated spread is curtailed along roadways first, with special focus on newly introduced populations like that at Nipomo, CA, which have not yet expanded. Future introductions should be expected, necessitating further investigation as new localities are discovered. Additionally, future research focusing on phenotypic plasticity is needed to reveal the strategies that enable Sahara mustard to invade multiple environments despite low genetic diversity.

## CONFLICT OF INTEREST

None declared.

## AUTHOR CONTRIBUTIONS

D.E.W. conceived of and designed the project with help from T.E.H., K.J.C., and J.D.G. Additionally, D.E.W. and K.J.C. carried out field and laboratory work. D.E.W., K.J.C., and O.F. performed analyses and drafted the manuscript with T.E.H. All authors contributed to writing the final version of the manuscript.

## Data Availability

Molecular data used in these analyses are available as a NCBI's sequence read archive (BioProject for B. tournefortii: PRJNA534338), subject to a 1‐year embargo period postpublication.
