# Supplementary material for: Multiple introductions and population structure during the rapid expansion of the invasive Sahara mustard (Brassica tournefortii)
Source: Ecol Evol. 2019 Jun 28;9(14):7928–41. doi: 10.1002/ece3.5239 (PMC6662425; doi:10.1002/ece3.5239)
Supplement: Supplementary file 1 [file ECE3-9-7928-s001.docx]

**Supplemental Information for:**

**Multiple introductions and population structure during the rapid expansion of the invasive Sahara mustard (*Brassica tournefortii*)**

Daniel E. Winkler, Kenneth J. Chapin, Olivier François,

J. David Garmon, Brandon S. Gaut, Travis E. Huxman

**Table of Contents:**

| **Table S1** | Page 2 |
| --- | --- |
| **Table S2** | Page 5 |
| **Figure S1** | Page 9 |
| **Video S1 legend** | Page 10 |

Table S1. Sampling location and site characteristics.

| **#** | **Site** | **location** | ***n*** | **latitude** | **longitude** | **Elevation (m)** | **ecoregion** |
| --- | --- | --- | --- | --- | --- | --- | --- |
| 1 | Irvine | South CA Coast | 20 | 33.63791 | -117.969 | -13.33 | California South Coast |
| 2 | Anza1 | Anza Borrego | 11 | 33.21406 | -116.466 | 1026.09 | California South Coast |
| 3 | Anza2 | Anza Borrego | 12 | 33.30025 | -116.387 | 223.15 | Sonoran Desert |
| 4 | Salton City | Imperial Valley | 12 | 33.2687 | -115.957 | -27.75 | Sonoran Desert |
| 5 | El Centro | Imperial Valley | 17 | 32.82803 | -115.5 | -11.65 | Sonoran Desert |
| 6 | Ocotillo | Imperial Valley | 19 | 32.64709 | -116.106 | 905.68 | California South Coast |
| 7 | San Diego | South CA Coast | 9 | 32.72335 | -117.138 | 41.22 | California South Coast |
| 8 | Coachella | Coachella Valley | 12 | 33.77248 | -116.304 | 24.64 | Sonoran Desert |
| 9 | Glamis | West AZ | 20 | 32.97718 | -115.171 | 82.8 | Sonoran Desert |
| 10 | Blythe | West AZ | 20 | 33.56059 | -114.657 | 70.54 | Sonoran Desert |
| 11 | Parker | West AZ | 19 | 33.99411 | -114.216 | 195.51 | Sonoran Desert |
| 12 | JOTR1 | Joshua Tree | 19 | 34.0488 | -115.217 | 450.5 | Sonoran Desert |
| 13 | JOTR2 | Joshua Tree | 19 | 34.13515 | -116.137 | 771.34 | Mojave Desert |
| 14 | MOJA1 | Mojave | 18 | 34.82923 | -116.694 | 590.13 | Mojave Desert |
| 15 | Ibis | West AZ | 13 | 35.07073 | -114.828 | 611.76 | Mojave Desert |
| 16 | Leeds | Northern | 7 | 37.22532 | -113.406 | 985.23 | Mojave Desert |
| 17 | MormonPk | Northern | 19 | 36.83129 | -114.373 | 850.17 | Mojave Desert |
| 18 | LAKE1 | Northern | 20 | 36.01262 | -114.731 | 466.12 | Mojave Desert |
| 19 | Amargosa | Northern | 18 | 36.56371 | -116.123 | 871.79 | Mojave Desert |
| 20 | Beatty | Northern | 13 | 36.85232 | -116.756 | 961.68 | Mojave Desert |
| 21 | Las Vegas | Northern | 11 | 36.0898 | -115.233 | 726.88 | Mojave Desert |
| 22 | MOJA2 | Mojave | 9 | 35.13412 | -116.207 | 457.71 | Mojave Desert |
| 23 | MOJA3 | Mojave | 10 | 35.26009 | -116.068 | 290.92 | Mojave Desert |
| 24 | MOJA4 | Mojave | 14 | 34.80259 | -115.612 | 1193.11 | Mojave Desert |
| 25 | Aguila | West AZ | 7 | 33.94289 | -113.161 | 667.28 | Sonoran Desert |
| 26 | Phoenix1 | Phoenix | 18 | 33.80308 | -112.24 | 501.69 | Sonoran Desert |
| 27 | Phoenix2 | Phoenix | 10 | 33.39348 | -111.662 | 429.83 | Sonoran Desert |
| 28 | Tortilla Flat | Apache Highlands | 9 | 33.52706 | -111.388 | 549.03 | Sonoran Desert |
| 29 | Roosevelt | Apache Highlands | 19 | 33.64701 | -111.112 | 824.45 | Sonoran Desert |
| 30 | Fort Apache | Apache Highlands | 19 | 33.79879 | -110.507 | 1053.48 | Apache Highlands |
| 31 | Las Cruces | New Mexico | 12 | 32.18376 | -106.678 | 1195.52 | Chihuahuan Desert |
| 32 | Fort Hancock | Texas | 11 | 31.29882 | -105.832 | 1114.53 | Chihuahuan Desert |
| 33 | El Paso | Texas | 15 | 31.75388 | -106.348 | 1182.78 | Chihuahuan Desert |
| 34 | Dragoon | East AZ | 16 | 32.08114 | -110.051 | 1502.66 | Apache Highlands |
| 35 | SAGU1 | Tucson | 11 | 32.17711 | -110.739 | 962.64 | Sonoran Desert |
| 36 | SAGU2 | Tucson | 18 | 32.32543 | -111.122 | 717.74 | Sonoran Desert |
| 37 | Malibu | Central Coast | 12 | 34.0221 | -118.809 | 50.84 | California South Coast |
| 38 | Nipomo | Central Coast | 12 | 35.04823 | -120.512 | 128.22 | California Central Coast |
| 39 | Chaney Ranch | Central Valley | 14 | 36.6231 | -120.609 | 188.78 | Great Central Valley |
| 40 | Murray | Central Valley | 11 | 36.07389 | -120.103 | 189.74 | California Central Coast |
| 41 | Bakersfield | Central Valley | 14 | 35.29217 | -118.752 | 272.18 | Great Central Valley |
| 42 | Victorville | Mojave | 16 | 34.50634 | -117.597 | 1035.46 | Mojave Desert |
| 43 | JOTR3 | Joshua Tree | 13 | 33.91534 | -115.829 | 561.53 | Mojave Desert |
| 44 | Palm Springs | Coachella | 18 | 33.92079 | -116.71 | 437.52 | Sonoran Desert |
| 45 | Anza3 | Anza Borrego | 13 | 33.13015 | -116.312 | 363.5 | California South Coast |
| 46 | DEVA | Northern | 14 | 35.90607 | -116.641 | -116.6 | Mojave Desert |
| 47 | Dateland | South AZ | 13 | 32.80141 | -113.541 | 130.86 | Sonoran Desert |
| 48 | Gila Bend | South AZ | 19 | 32.93566 | -112.68 | 244.3 | Sonoran Desert |
| 49 | Rocky Pt | South AZ | 15 | 32.60002 | -112.871 | 346.92 | Sonoran Desert |
| 50 | ORPI1 | South AZ | 17 | 32.02349 | -112.802 | 522.6 | Sonoran Desert |
| 51 | ORPI2 | South AZ | 9 | 32.00967 | -112.709 | 697.56 | Sonoran Desert |
| 52 | ORPI3 | South AZ | 12 | 32.13016 | -112.768 | 556.48 | Sonoran Desert |

Table S2. Summary statistics for each sampling site. *H*_o_ (± SE) = the observed heterozygosity for SNPs, *H*_e_ (± SE) = the expected heterozygosity for SNPs, *F_is_* = index of fixation, *S* = selfing rate, *T* = outcrossing rate, and private = the number of private alleles. Standard errors are reported parenthetically.

| location | *H*_o_ (± SE) | *H*_e_ (± SE) | *F_is_* | *S* | *T* | private |
| --- | --- | --- | --- | --- | --- | --- |
| Irvine | 0.055 (±0.0063) | 0.1301 (± 0.0053) | 0.8363 | 0.9109 | 0.0891 | 1 |
| Anza1 | 0.058 (±0.0067) | 0.0997 (± 0.0046) | 0.8535 | 0.9210 | 0.0790 | 0 |
| Anza2 | 0.0587 (±0.0066) | 0.1091 (± 0.0047) | 0.8394 | 0.9127 | 0.0873 | 0 |
| Salton City | 0.0576 (±0.0062) | 0.1234 (± 0.0053) | 0.8433 | 0.9150 | 0.0850 | 0 |
| El Centro | 0.0613 (±0.0065) | 0.1071 (± 0.0047) | 0.8429 | 0.9148 | 0.0852 | 0 |
| Ocotillo | 0.0556 (±0.0065) | 0.0504 (± 0.0038) | 0.8469 | 0.9171 | 0.0829 | 0 |
| San Diego | 0.0588 (±0.0064) | 0.1171 (± 0.0051) | 0.8539 | 0.9212 | 0.0788 | 0 |
| Coachella | 0.0553 (±0.0064) | 0.0754 (± 0.0039) | 0.7332 | 0.8461 | 0.1539 | 0 |
| Glamis | 0.0557 (±0.0066) | 0.0767 (± 0.0039) | 0.8301 | 0.9072 | 0.0928 | 0 |
| Blythe | 0.0556 (±0.0066) | 0.0359 (± 0.0038) | 0.8305 | 0.9074 | 0.0926 | 1 |
| Parker | 0.0547 (±0.0065) | 0.0356 (± 0.0038) | 0.7854 | 0.8798 | 0.1202 | 0 |
| JOTR1 | 0.0576 (±0.0066) | 0.0765 (± 0.004 | 0.8342 | 0.9096 | 0.0904 | 0 |
| JOTR2 | 0.0564 (±0.0066) | 0.0369 (± 0.0039) | 0.8222 | 0.9024 | 0.0976 | 0 |
| MOJA1 | 0.0584 (±0.0068) | 0.0373 (± 0.0038) | 0.8434 | 0.9150 | 0.0850 | 1 |
| Ibis | 0.058 (±0.0068) | 0.0374 (± 0.0039) | 0.8397 | 0.9129 | 0.0871 | 1 |
| Leeds | 0.0577 (±0.0067) | 0.0864 (± 0.0039) | 0.8620 | 0.9259 | 0.0741 | 1 |
| MormonPk | 0.0588 (±0.0067) | 0.0775 (± 0.004) | 0.8343 | 0.9096 | 0.0904 | 0 |
| LAKE1 | 0.0547 (±0.0064) | 0.0891 (± 0.0039) | 0.8354 | 0.9103 | 0.0897 | 0 |
| Amargosa | 0.0561 (±0.0065) | 0.0778 (± 0.004) | 0.8419 | 0.9142 | 0.0858 | 1 |
| Beatty | 0.0586 (±0.0066) | 0.0926 (± 0.004) | 0.8400 | 0.9130 | 0.0870 | 0 |
| Las Vegas | 0.0586 (±0.0066) | 0.0716 (± 0.004) | 0.8544 | 0.9215 | 0.0785 | 2 |
| MOJA2 | 0.0589 (±0.0066) | 0.0925 (± 0.0039) | 0.8492 | 0.9185 | 0.0815 | 2 |
| MOJA3 | 0.0566 (±0.0064) | 0.0833 (± 0.0039) | 0.8443 | 0.9156 | 0.0844 | 1 |
| MOJA4 | 0.0599 (±0.0065) | 0.0894 (± 0.004) | 0.8345 | 0.9098 | 0.0902 | 0 |
| Aguila | 0.0543 (±0.0063) | 0.0884 (± 0.0038) | 0.8609 | 0.9252 | 0.0748 | 0 |
| Phoenix1 | 0.0582 (±0.0066) | 0.0902 (± 0.004) | 0.8327 | 0.9087 | 0.0913 | 2 |
| Phoenix2 | 0.0541 (±0.0063) | 0.0892 (± 0.0038) | 0.8585 | 0.9239 | 0.0761 | 2 |
| Tortilla Flat | 0.0566 (±0.0067) | 0.0365 (± 0.0039) | 0.8558 | 0.9223 | 0.0777 | 3 |
| Roosevelt | 0.057 (±0.0067) | 0.0369 (± 0.0038) | 0.7933 | 0.8847 | 0.1153 | 0 |
| Fort Apache | 0.0546 (±0.0065) | 0.036 (± 0.0038) | 0.8416 | 0.9140 | 0.0860 | 1 |
| Las Cruces | 0.0556 (±0.0066) | 0.0358 (± 0.0038) | 0.8453 | 0.9161 | 0.0839 | 2 |
| Fort Hancock | 0.0557 (±0.0066) | 0.0363 (± 0.0038) | 0.8414 | 0.9139 | 0.0861 | 2 |
| El Paso | 0.0555 (±0.0066) | 0.0355 (± 0.0038) | 0.8405 | 0.9134 | 0.0866 | 2 |
| Dragoon | 0.0576 (±0.0067) | 0.0368 (± 0.0038) | 0.8399 | 0.9130 | 0.0870 | 1 |
| SAGU1 | 0.0548 (±0.0066) | 0.0356 (± 0.0038) | 0.8493 | 0.9185 | 0.0815 | 2 |
| SAGU2 | 0.0541 (±0.0065) | 0.035 (± 0.0038) | 0.8383 | 0.9120 | 0.0880 | 0 |
| Malibu | 0.055 (±0.0064) | 0.0361 (± 0.0037) | 0.8521 | 0.9201 | 0.0799 | 1 |
| Nipomo | 0.0551 (±0.0066) | 0.0356 (± 0.0038) | 0.7865 | 0.8805 | 0.1195 | 303 |
| Chaney Ranch | 0.0553 (±0.0065 | 0.0362 (± 0.0038) | 0.8462 | 0.9167 | 0.0833 | 0 |
| Murray | 0.0554 (±0.0065) | 0.0367 (± 0.0038) | 0.8508 | 0.9194 | 0.0806 | 0 |
| Bakersfield | 0.0531 (±0.0065) | 0.0341 (± 0.0038) | 0.8358 | 0.9106 | 0.0894 | 1 |
| Victorville | 0.0581 (±0.0068) | 0.0368 (± 0.0039) | 0.8397 | 0.9129 | 0.0871 | 1 |
| JOTR3 | 0.058 (±0.0066) | 0.0375 (± 0.0038) | 0.8456 | 0.9164 | 0.0836 | 1 |
| Palm Springs | 0.0589 (±0.0067) | 0.0767 (± 0.004) | 0.8189 | 0.9004 | 0.0996 | 1 |
| Anza3 | 0.059 (±0.0066) | 0.0795 (± 0.0041) | 0.8435 | 0.9151 | 0.0849 | 0 |
| DEVA | 0.0626 (±0.0067) | 0.0895 (± 0.0043) | 0.8385 | 0.9122 | 0.0878 | 1 |
| Dateland | 0.059 (±0.0066) | 0.0761 (± 0.004) | 0.8454 | 0.9162 | 0.0838 | 0 |
| Gila Bend | 0.0572 (±0.0062) | 0.1434 (± 0.0058) | 0.8056 | 0.8923 | 0.1077 | 3 |
| Rocky Pt | 0.0608 (±0.0063) | 0.1117 (± 0.0048) | 0.8379 | 0.9118 | 0.0882 | 3 |
| ORPI1 | 0.0792 (±0.0063) | 0.1154 (± 0.005) | 0.8451 | 0.9161 | 0.0839 | 0 |
| ORPI2 | 0.06 (±0.0066) | 0.0474 (± 0.0038) | 0.8499 | 0.9189 | 0.0811 | 1 |
| ORPI3 | 0.0637 (±0.0065) | 0.0773 (± 0.0042) | 0.8459 | 0.9165 | 0.0835 | 0 |

#


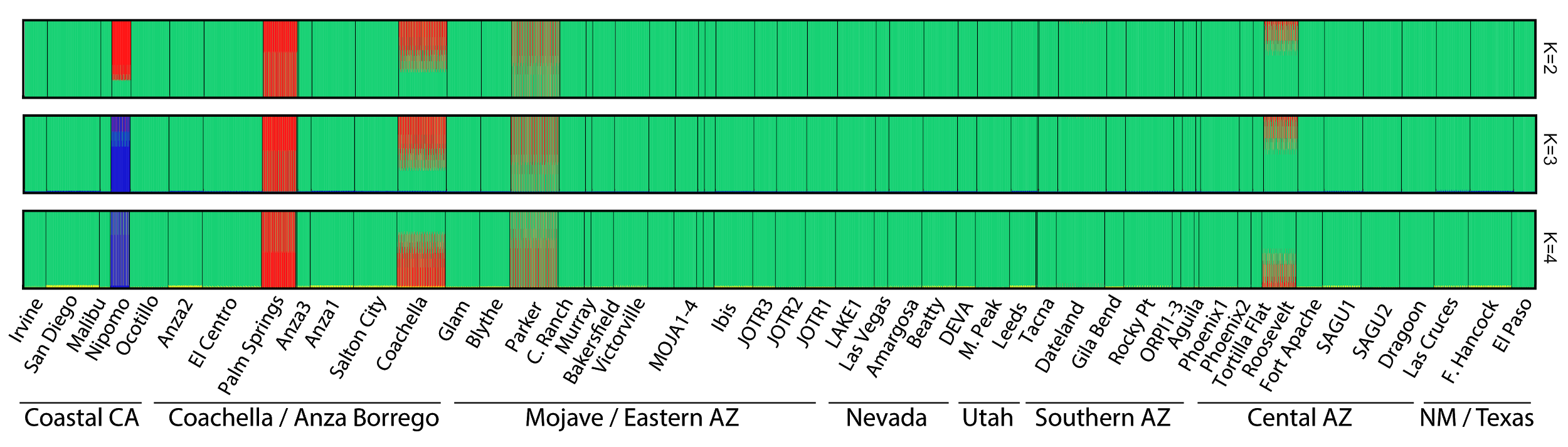


**Figure S1.** Individual assignments from TESS analyses based on 1,525 SNP loci of 744 individuals from across the invaded range of Sahara mustard (*Brassica tournefortii*). Barplots are averaged across ten runs for *K* = 2–4.

**Video S1.** Active map of Sahara mustard (*Brassica tournefortii*) spread using herbaria samples collected annual since the first collection in 1927 until 2013 when the present study commenced. Points are color-coded by decade and trace the movement of the species in California, Arizona, Nevada, Utah, and northwestern Mexico. Collection biases are highlighted by rapid roadway sampling that occurred in 2004–2005 (https://www.cal-ipc.org/solutions/research/saharan/).
